# Diagnosis of cerebral microbleed via VGG and extreme learning machine trained by Gaussian map bat algorithm

**DOI:** 10.1007/s12652-020-01789-3

**Published:** 2020-02-24

**Authors:** Siyuan Lu, Kaijian Xia, Shui-Hua Wang

**Affiliations:** 1 School of Informatics, University of Leicester, Leicester LE1 7RH, UK; 2 The Affiliated Changshu Hospital of Soochow University (Changshu No. 1 People’s Hospital), Changshu 215500, Jiangsu, China; 3 School of Computer Science and Engineering, Changshu Institute of Technology, Changshu 215500, Jiangsu, China

**Keywords:** Cerebral microbleed, Magnetic resonance image, Deep learning, Extreme learning machine, Gaussian map

## Abstract

Cerebral microbleed (CMB) is a serious public health concern. It is associated with dementia, which can be detected with brain magnetic resonance image (MRI). CMBs often appear as tiny round dots on MRIs, and they can be spotted anywhere over brain. Therefore, manual inspection is tedious and lengthy, and the results are often short in reproducible. In this paper, a novel automatic CMB diagnosis method was proposed based on deep learning and optimization algorithms, which used the brain MRI as the input and output the diagnosis results as CMB and non-CMB. Firstly, sliding window processing was employed to generate the dataset from brain MRIs. Then, a pre-trained VGG was employed to obtain the image features from the dataset. Finally, an ELM was trained by Gaussian-map bat algorithm (GBA) for identification. Results showed that the proposed method VGG-ELM-GBA provided better generalization performance than several state-of-the-art approaches.

## 1 Introduction

Cerebral microbleeds (CMBs) appear as tiny round black dots with a diameter of 5–10 mm on brain magnetic resonance image (MRI), which are caused by microvascular diseases. Typically, CMBs can be found in deep brain and corticosubcortical regions. Inspection of CMB with naked eye is difficult and time-consuming for doctors and radiologists and the diagnosis results are prone to suffer from high inter-observe variance, which means that different experts may come up with different diagnosis results. Hence, it is significant and urgent to develop automatic CMB diagnosis system which can provide a reference for doctors to make decisions and help improve the efficiency of whole medical system. Recently, the rapid development of deep learning and computer vision achieved substantial progress and those advanced algorithms and models have been successfully applied in many practical problems, such as face recognition, computer aided diagnosis, and automatic driving. Therefore, many researchers are trying to use artificial intelligence for automated and accurate CMB diagnosis. The diagnosis of CMB is to label every pixel as CMB or non-CMB.

Barnes et al. (2011) proposed a semi-automated CMB diagnosis method. Firstly, hypointensities were generated by statistic thresholding algorithm. Then, the true CMBs were classified by support vector machine (SVM) from the hypointensities. Their method achieved sensitivity of 81.7% and worked faster than several alternatives. Kuijf et al. (2012) suggested to employ radial symmetry transform (RST) to identify potential CMB. The transformed images were checked by human for evaluation. The proposed method yielded sensitivity of 71.2% and required less time and effort of manual checking. Bian et al. (2013) utilized 2D fast RST to generate potential CMBs and eliminated the false ones by geometry features. de Bresser et al. (2013) analyzed the challenges and the reliability of image processing in diagnosis of CMB. Fazlollahi et al. (2015) leveraged multi-scale Laplacian approach to generate CMB candidates and extracted shape features from these candidates. Finally, a cascade random forest was trained to distinguish true CMB from false ones. They achieved sensitivity of 87% on their possible and definite dataset. Zhang et al. (2017) introduced deep learning methods for CMB diagnosis. A seven-layer deep neural network (DNN) was constructed and trained on their CMB dataset for classification.

From the above research, we can find that current diagnosis of CMB usually follows the flow of feature extraction, classifier training and classification. Feature extraction is used to generate some features and representations from brain MRIs to form the feature vector. It is a necessary step as MRIs are of high volume and contain massive information, which occupy too much memory and increase the computational complexity during classifier training. The classifiers used are supervised machine learning algorithms which employ the image features as input and image labels as the expected output in training. In the final classification stage, the trained classifiers were tested on test sets to evaluate their classification performance. The current research has achieved good results, but there are still some problems existing. RST was often employed to extract features, but it belongs to a domain dependent method. The features obtained by RST may work only on certain datasets but fail on others. Deep learning models are powerful in image recognition, but they are often trained on datasets with millions of samples. Hence, it may be inappropriate to directly use CMB datasets to train DNNs, which usually contain only thousands of samples. Overfitting is likely to happen in those models, which means the classifiers can achieve high accuracy on training set but low accuracy on testing set.

To overcome these problems, a novel cerebral microbleed (CMB) diagnosis method based on VGG, extreme learning machine (ELM) and Gaussian map bat algorithm was proposed in this paper. VGG is a famous convolutional neural network (CNN) model, which was employed for extracting features from brain MRIs. Instead of training VGG with our CMB dataset, we just directly used a pre-trained VGG to generate image features. Then, the feature vectors were fed into an ELM for training. ELM is a learning algorithm for single hidden layer feedforward network (SLFN). Unlike back propagation algorithm, ELM trains the network in only three steps without gradient-descent based iterations. So, ELM converges thousand times faster than back propagation algorithm and yields good classification performance at the same time. Gaussian-map bat algorithm (GBA) was leveraged to further improve the ELM’s generalization ability. With all these successful components, our CMB diagnosis method achieved good results, which outperformed state-of-the-art approaches.

The rest of this paper is organized as follows. The related work is presented in Sect. 1. Section 2 explains the CMB datasets. Detailed explanation and analysis are in Sect. 3, including: VGG, ELM and GBA. We introduced three different chaotic maps for Bat algorithms. Section 4 discusses the experiment environment and settings. Section 5 gives the experiment results and discussion. Finally, Sect. 6 presents the conclusion and future research plan.

## 2 Related work

Currently, CMB detection methods can be classified into two categories: classifier learning and feature learning. The classifier learning generator focuses on the training and optimization of classifier and uses handcraft image features. Hong and Lu (2019) proposed a CMB detection system combined back-propagation neural network (BPNN) with discrete wavelet transform (DWT). DWT was for feature extraction and BPNN served as classification algorithm. Principle component analysis (PCA) was employed to reduce the feature number. Ourselin et al. (2015) firstly performed multiple RST to obtain CMB candidates. Then, the 3D patches were used to form the feature vectors. Finally, random forest regression was selected as the classification algorithm. Experimental results suggested that their method achieved sensitivity of 85.7%. Tao and Cloutie (2018) utilized genetic algorithm (GA) to train the parameters in BPNN for identification of CMB images. van den Heuvel et al. (2016) proposed a method to detect CMB in patients with traumatic brain injury. Firstly, twelve features were extracted from each voxel and a random forest was leveraged to predict the CMB candidates’ locations. Then, a classifier based on object was trained to distinguish true CMB from blood vessels. They also developed user-friendly interface for experts. Gagnon (2017) used Naive Bayesian classifier (NBC) to detect CMB. Their accuracy achieved 76.90%. Zhang et al. (2017) used single hidden layer feedforward neural network with scaled conjugate gradient to detect CMB in cerebral autosomal-dominant arteriopathy with subcortical infarcts and Leukoencephalopathy (CADASIL) patients. They compared the performance of their systems of three different activation functions: Logistic Sigmoid, rectified linear unit (ReLU) and leaky rectified linear unit (LReLU). Experimental results showed that LReLU achieved sensitivity of 93.05%, which was the best of the three activation functions. Qian (2018) employed cat swarm optimization to handle brain diseases.

On the other hand, feature learning is playing a more and more important role in machine learning and computer vision tasks with the deep CNN models. Feature extraction and reduction is inevitable for image classification problems, because the images contain too much information that the excessive features have bad effect on the classifier training. CNN provides a general framework for image feature learning. With convolution operation, features can be learned automatically compared with fully connected networks, convolution also reduces the parameters in CNN and implements weight sharing. Chen et al. (2018) designed a 3D residual neural network to diagnose CMB in 3D brain MRIs. Hong et al. (2019) used ResNet to detect CMB. Transfer learning was leveraged to train the ResNet on CMB dataset, which helps to improve the generalization ability of the network.

Therefore, we want to combine the merits of both feature learning and classifier learning. For feature learning, a pretrained VGG was employed as the feature extractor, and for classifier learning, we proposed a novel Gaussian-map bat algorithm to optimize extreme learning machine. In this way, the time consuming training of deep CNN can be avoided and better classification performance can be achieved with optimization of classifier parameters.

## 3 Material

### 3.1 Data acquisition

Totally, ten CADASIL samples and ten healthy controls were obtained, with each in size of 364 × 448 × 48 pixels. All the images were reconstructed on Syngo MR B17 software and labeled by three experts over 10 years’ experience from Nanjing medical university. We disregarded the microvessels and lesions of over 10 mm diameters. The voxels of both possible and definite CMB were included in CMB category in our experiment.

### 3.2 Image pre-processing

**Fig. 1:**
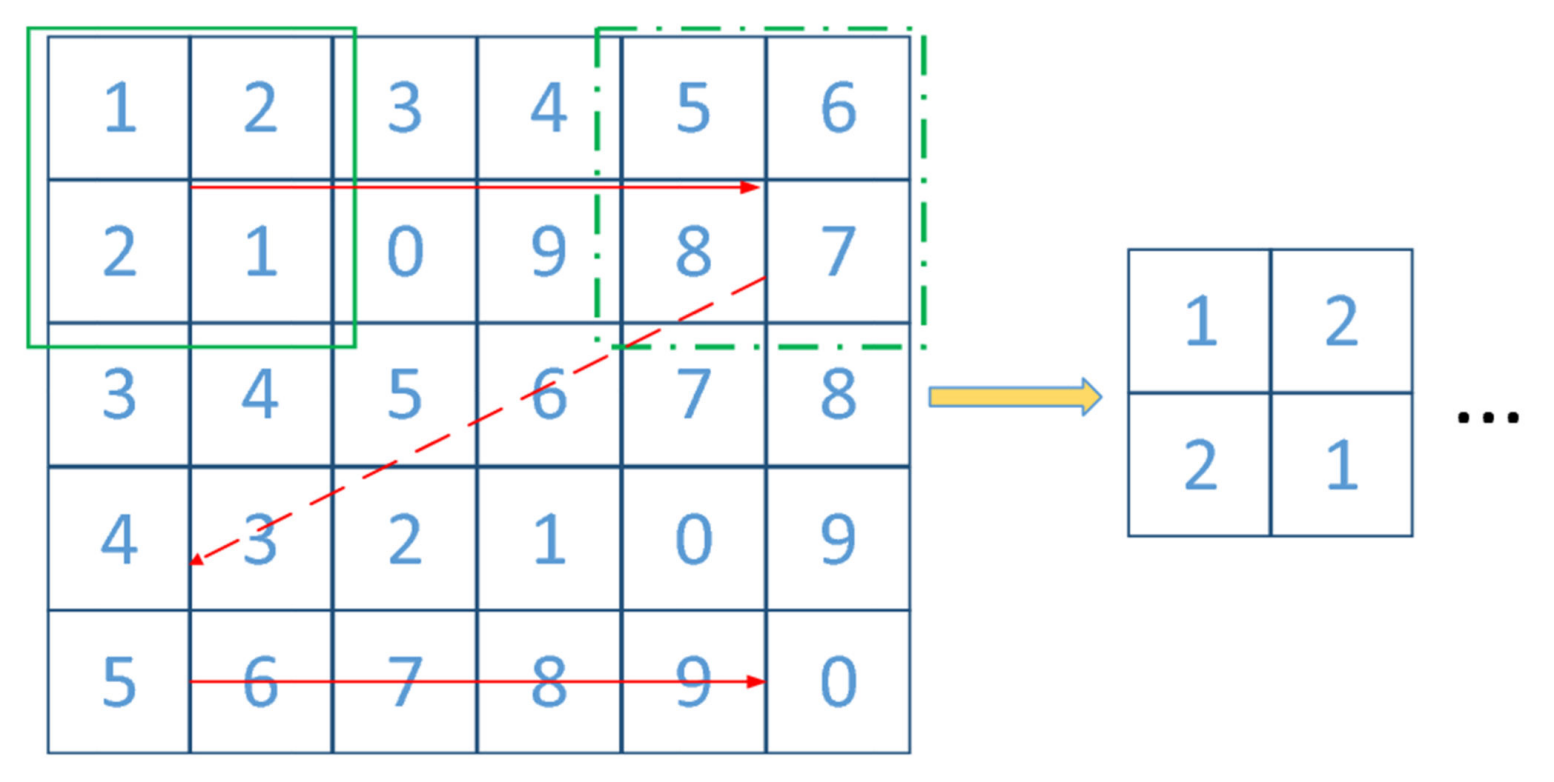
Sliding neighborhood processing

**Fig. 2:**
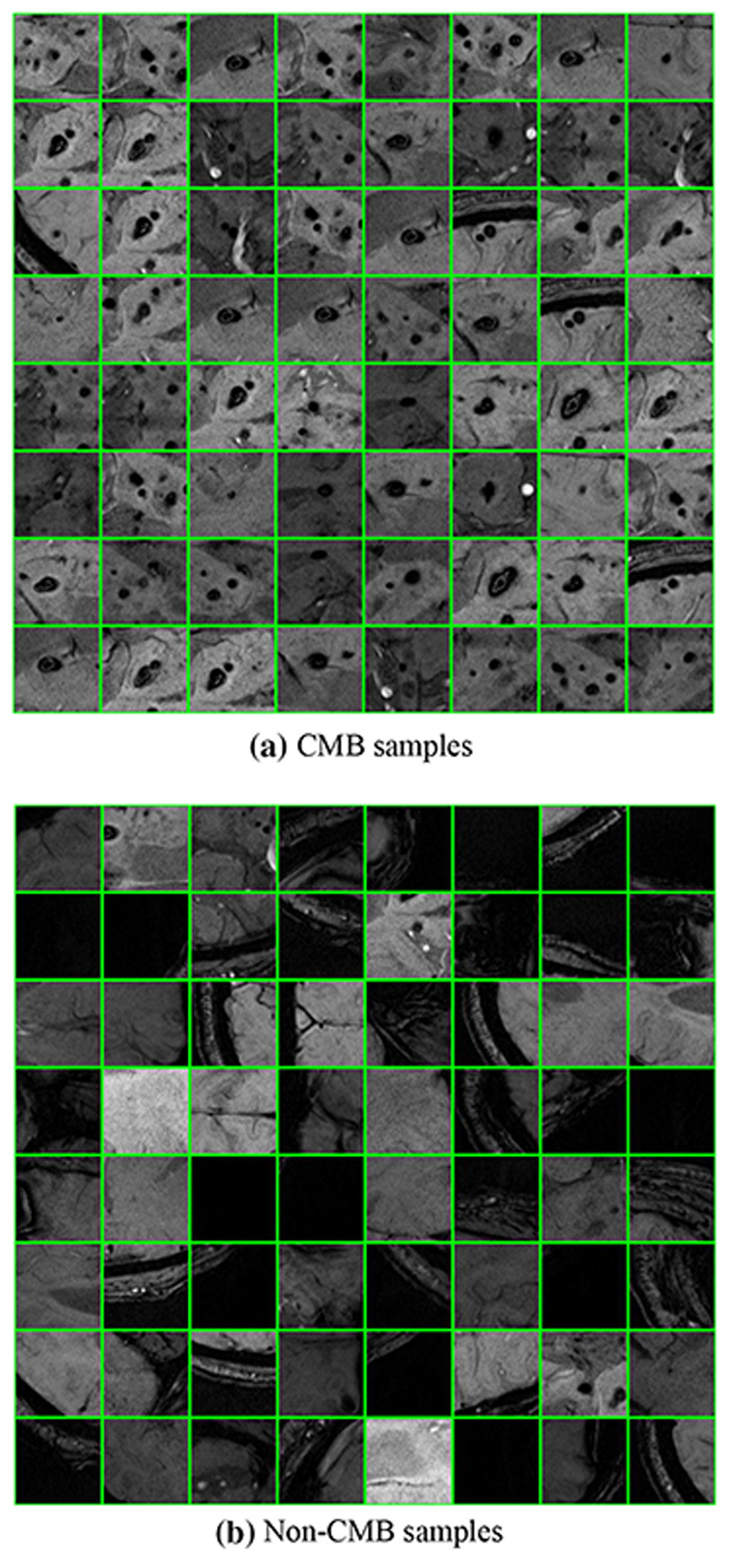
Dataset samples **a** CMB samples. **b** Non-CMB samples

It is not suitable to directly use the ten CADASIL samples and ten healthy controls to train the classifier. Meanwhile, it is meaningful to locate the CMB while classification. Hence, we proposed to leverage sliding neighborhood processing (SNP) for image pre-processing. As is shown in Fig. 1, SNP employed a window to move from the top left to the bottom right corner of the images to generate samples for training and testing of the classifier. The generated samples are in the same size of the window, and their locations can be obtained. The labels of these samples are dependent on their center voxels. If the center voxel of a sample is located within CMB region, it will be labeled as CMB, if not, it is a non-CMB sample. Figure 2 presents some samples in our dataset.

## 4 Methods

Our CMB diagnosis method followed the convention of image recognition, which contains feature extraction and classifier training. For feature extraction, a CNN model was used, which is called VGG. But we did not train it on our dataset. Instead, we simply extracted the output of certain layer in the pre-trained VGG to form the feature vector. Compared with various handcrafted image features, the features generated by CNN are more robust for classification, because CNN can learn features and representations automatically from low level to high level. The obtained feature vectors were then sent into an ELM for training and classification. We proposed to optimize the weight and bias of ELM with three BAC techniques to eliminate the side effect of randomness in ELM and improve its classification performance.

### 4.1 VGG

VGG is a famous CNN model proposed by Simonyan and Zisserman (2014). VGG won the ImageNet Large-Scale Visual Recognition Challenge (ILSVRC) in 2014, and the representations learned can be transferred to other image recognition tasks. By analysis and experiment, it can be revealed that the depth of CNN plays an important role for the representation learning ability of CNN, and that deeper models with more representation learning layers tend to achieve better accuracy than shallow models. 7 × 7 filters were replaced by 3 × 3 filters to achieve better discriminant ability. The depth of the model is increased while the number of total parameters remains at the same level. Multi-scale training was employed to improve the classification accuracy. VGG is easier to converge because the parameters in shallow layers are pre-initialized. In this study, we chose VGG-16 as the feature extractor, the detailed structural information is provided in Table 1. There are some other transfer learning techniques (Yu 2019; Govindaraj 2019) which we will test in the future studies.

There are totally 41 layers in VGG-16, but only 16 layers have trainable weights, including 13 convolution layers and 3 fully connected layers. Five pooling layers are also used in this model.

Convolution layers use filters to generate feature from images (Jiang 2019; Tang 2018; Pan 2018a, b). The filters are assigned with trainable weights. For an image *I* in size of (*W*, *H*) and a filter *F* in size of (*m,n*), the convolution expression is

(1) $$\;Convolution\;(I*F)(x,y)=\sum_{W}\sum_{H}I(x-m,y-n)F(m,n)$$

**Fig. 3:**
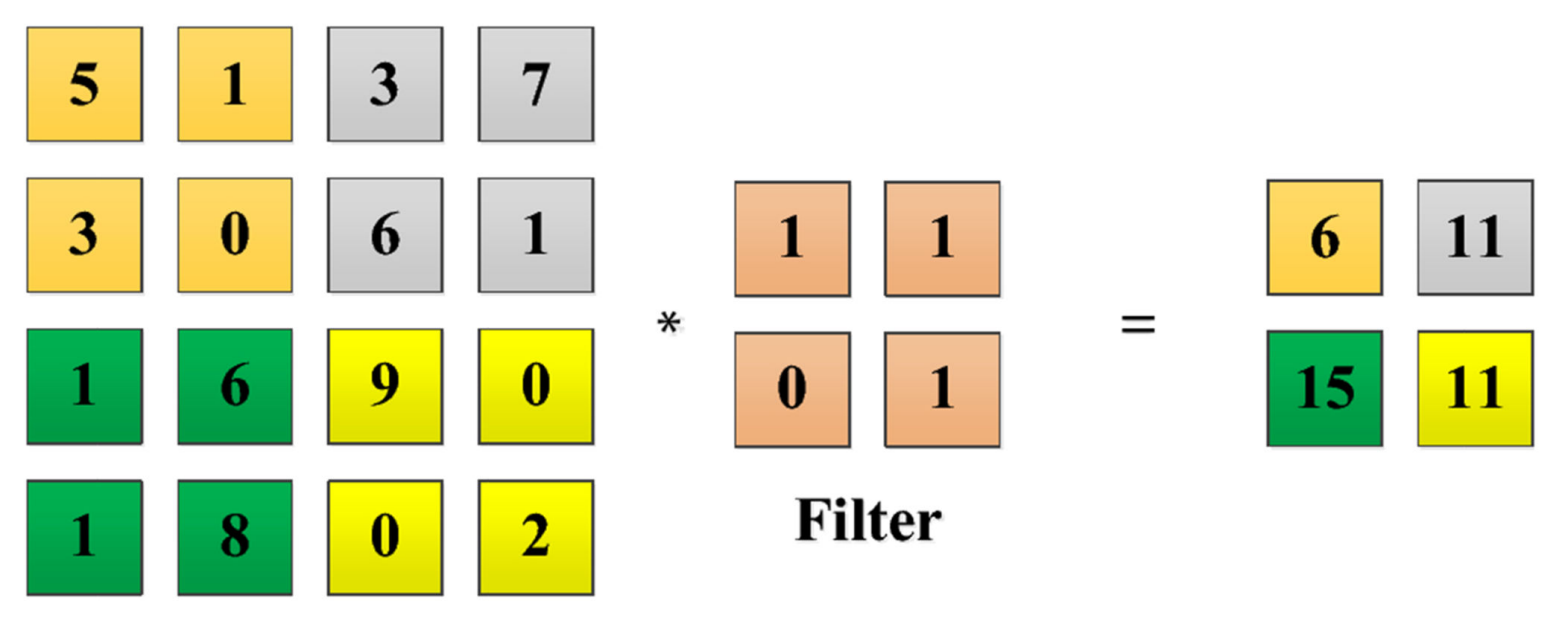
Convolution operation

**Fig. 4:**
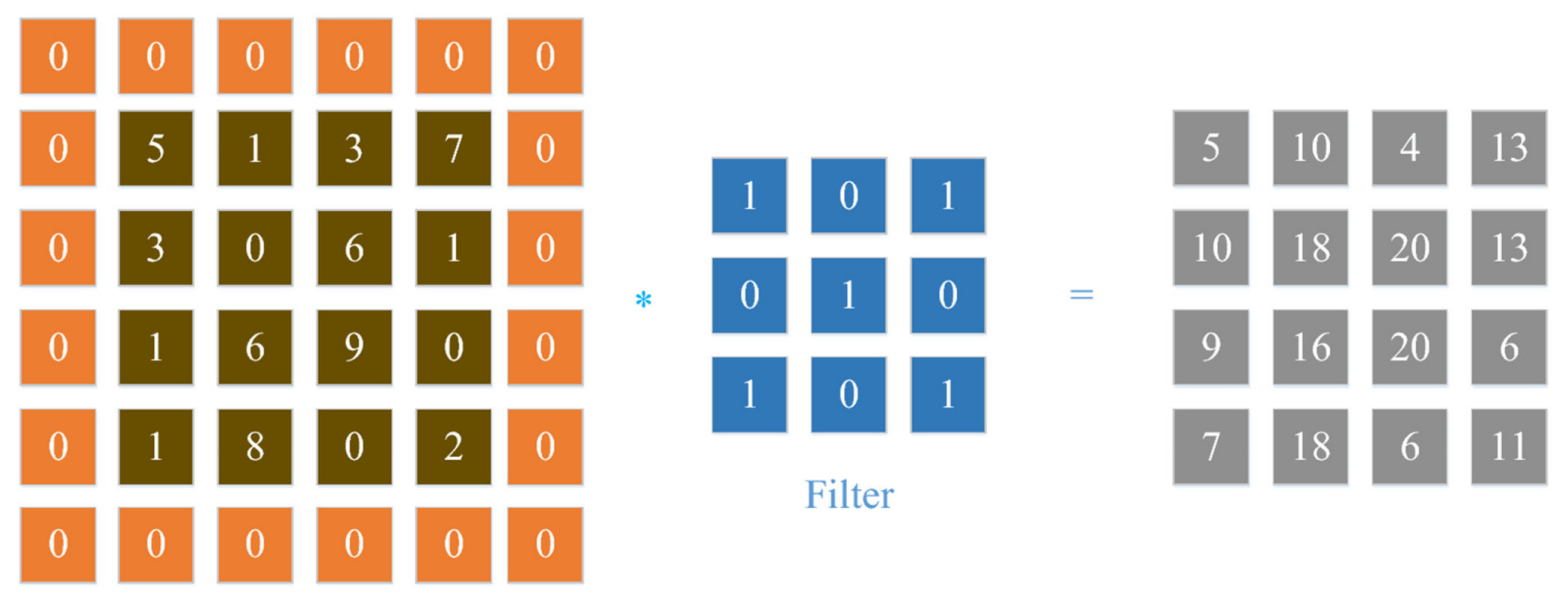
Convolution with zero padding

An example is given in Fig. 3, with a filter of 2 × 2 and stride 2. The size of obtained feature map is 2 × 2. It can be found that the size of feature map will shrink after each convolution which will inevitably cause information loss of image edges and corners. To keep the size of the feature maps, zero padding is often employed, which add pixels of zero intensity values around the edges of input image before convolutional. The relationship between the convolutional output feature map with the input image, the filter size and stride are given below:

(2) $$h_{\text{map}\;}=\frac{\left(h_{\text{input}\;}-h_{\text{filter}\;}+2\times p\right)}{s}+1$$

(3) $$w_{\text{map}\;}=\frac{\left(w_{\text{input}\;}-w_{\text{filter}\;}+2\times p\right)}{s}+1$$

where *w* and *h* denote the width and height, *p* represents the padding size, and *s* denotes the stride value. With appropriate filter size and zero padding size, the size of output feature size can be the same as that of input image. A toy example is shown in Fig. 4, with 3 × 3 filter and stride 1.

**Fig. 5:**
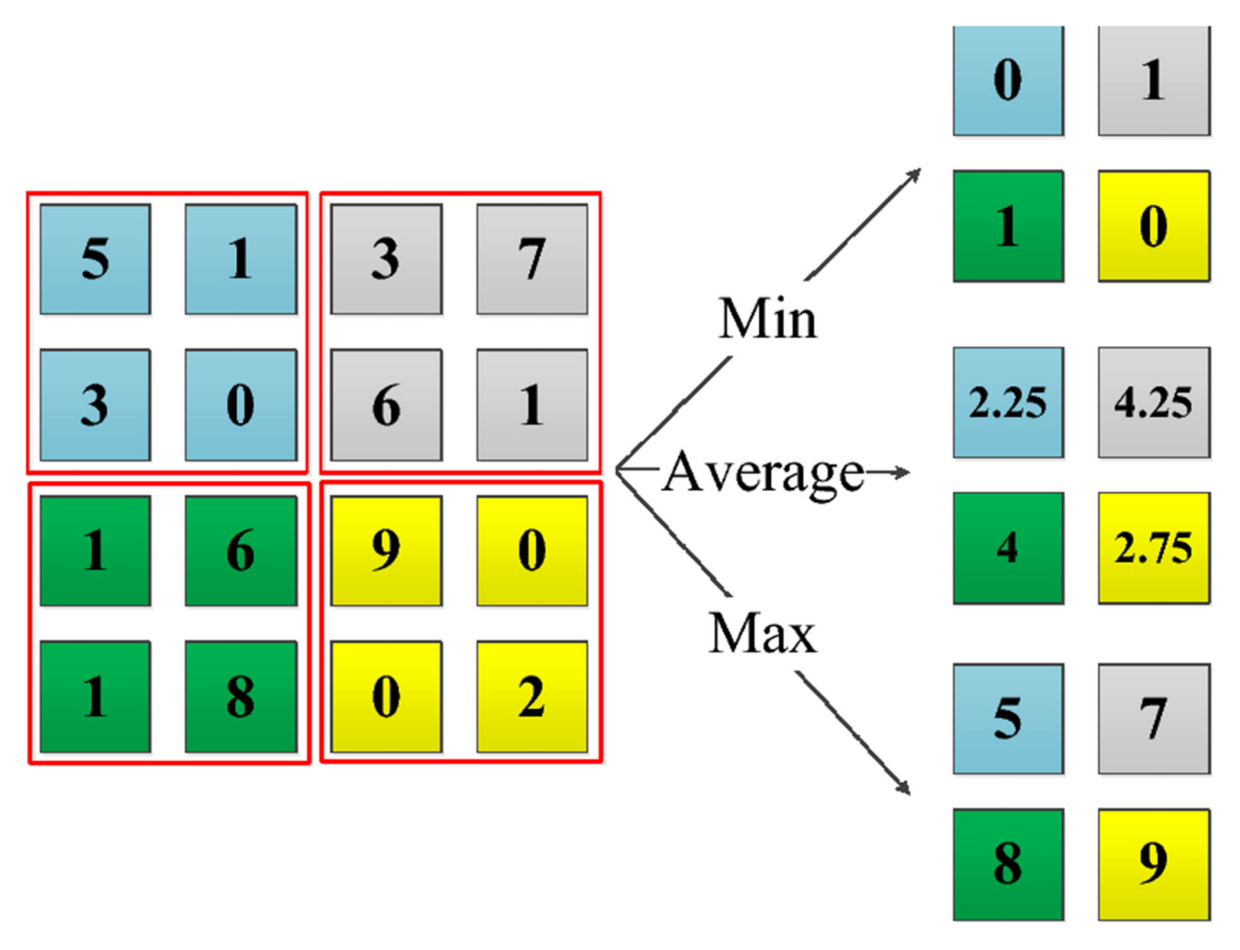
Pooling examples

The volume of feature maps can be much higher than that of the input image, so pooling layers are made for feature reduction. Pooling operation can maintain the outstanding features of the input feature maps while disregard the excessive features. In a pooling layer, a local perspective field is employed to extract features from the field by certain strategy, like max pooling and average pooling, as shown in Fig. 5.

The feature maps are finally vectorized and sent to fully connected layers. Fully connected layers are just like the structure of classical neural networks, every node is connected with all the nodes in adjacent layers with learnable weights. Fully connected layers are trained to classify the input features, so they usually appear at the rear of the CNN.

Deep learning was not widely applied until the recent 10 years, because of the gradient vanishing problem. As the layers get deeper, the gradient will approach zero, so the error cannot be propagated backward effectively. Activation function is an important factor of this problem. Traditional networks usually use sigmoid function as their activation function, as expressed below:

(4) $$Sigmoid(x)=\frac{1}{1-e^{-x}}$$

Sigmoid is popular because its gradient is easy to compute:

(5) Sigmoid′(x)=sigmoid(x)×[1−sigmoid(x)]

However, sigmoid does not work anymore in deep models. Rectified linear unit (ReLU) is often preferred in deep learning. ReLU is an easy function, if the input is positive, the output is the same value as the output. Otherwise, the output is zero:

(6) ReLU(x)=max(x,0)

The gradient value of ReLU function is always 1 if the input is positive, so the error can be propagated backward effectively. Additionally, in the last fully connected layer, the activation function is usually selected as softmax, because it can map the input to probability which is beneficial for classification. The softmax is defined as

(7) $$softmax(\text{x}{)}_{i}=\frac{\exp(x_{i})}{\sum_{j=1}^{n}\exp(x_{j})}$$

### 4.2 ELM

VGG is an effective model for image classification, but the diagnosis of CMB is a binary problem and our dataset is not big enough to train such a deep CNN. Overfitting is likely to happen. So, VGG is only employed for feature extraction, and for classification, a novel algorithm was selected, called ELM. ELM is a learning algorithm for SLFN, which is a classical neural network (Guang-Bin et al. 2006; Zhao 2018). The most famous training algorithm for classical network is back propagation (BP). However, the iteration of error backward is time-consuming, and the result obtained by BP is not ensured as the global best. ELM solves the training problem in another way. By random mapping in the input layer and pseudo inverse, ELM converges much fast than BP neural network. The performance of ELM is good as well. Hence, ELM has been applied to solve many practical problems, including image recognition (Zhang et al. 2017), prediction (Wei et al. 2019; Zou et al. 2017), clustering (Huang et al. 2018; Peng et al. 2016).

**Fig. 6:**
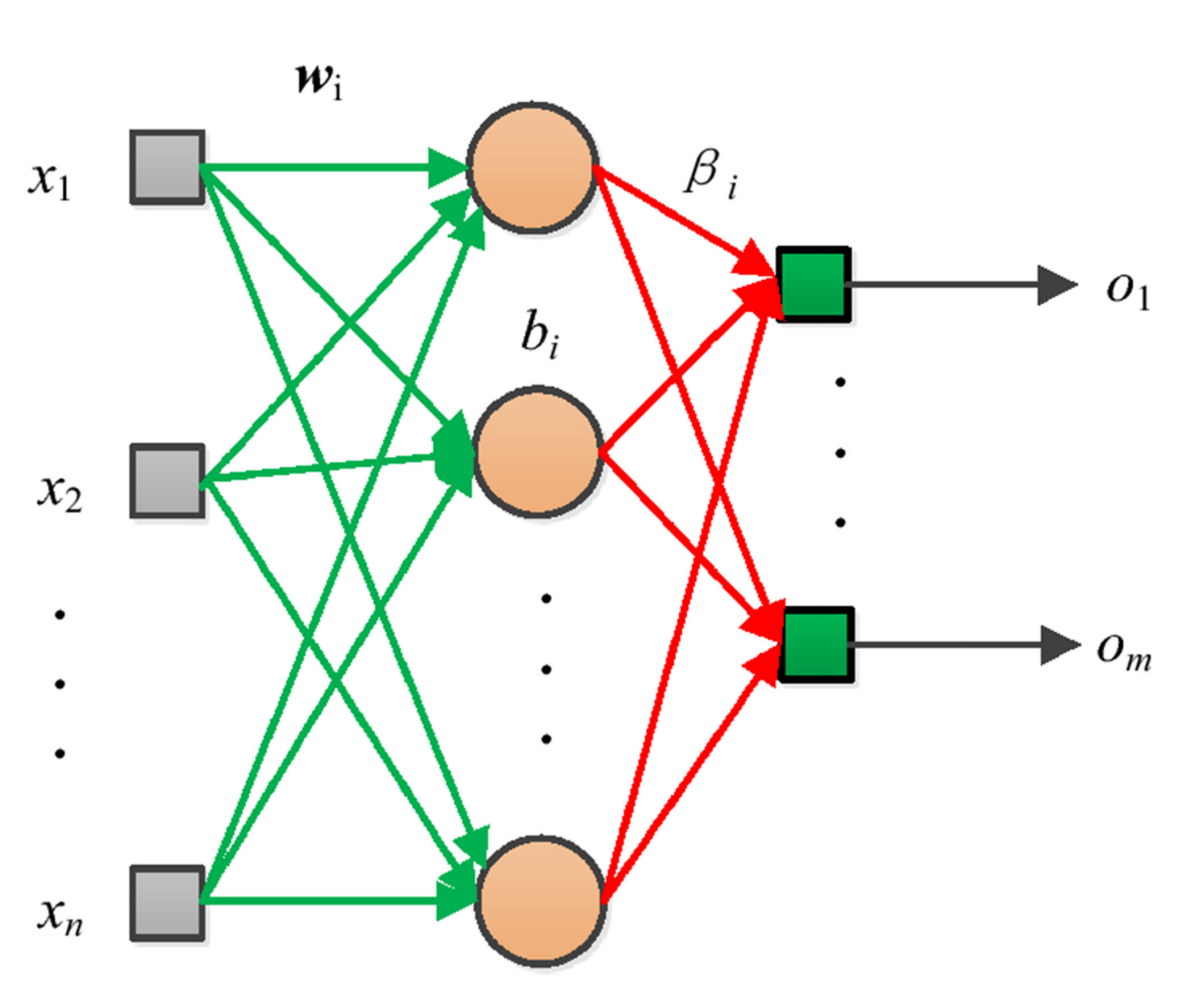
ELM

The structure of ELM is presented in Fig. 6, which is composed of three layers. Training of ELM is to determine the values of parameters, including hidden weight ***w***_i_, hidden bias *b*_i_ and output weight *β*_i_. The input ***x*** = (*x*_1_,*x*_2_,…,*x*_n_) and output ***o*** = (*o*_1_,*o*_2_,…,*o*_m_) are defined by the specific problems. Suppose a training dataset M:

(8) $$M=\{({\text{x}}_{i},{\text{t}}_{i})|{\text{x}}_{i}\in R^{n},{\text{t}}_{i}\in R^{m},i=1,\ldots ,N\}$$

where ***x**_i_* = (*x*_*i*1_,*x*_*i*2_, …,*x*_*in*_)^T^ ∈ *R^n^* denotes the input and ***t**_i_* = (*t*_*i*1_,*t*_*i*2_, …,*t*_*in*_)^T^ ∈ *R^m^* represents its label, and the ELM has $\hat{N}$ hidden nodes, then its output is:

(9) $$\sum_{i=1}^{\hat{N}}\beta_{i}f_{i}({\text{x}}_{j})=\sum_{i=1}^{\hat{N}}\beta_{i}f_{i}({\text{w}}_{i}{\text{x}}_{j}+b_{i})={\text{o}}_{j},j=1,\ldots ,\text{N}$$

where *f*(*x*) is the activation function in hidden layer. The training object is to make the output of ELM equals to the labels:

(10) $$\sum_{i=1}^{\hat{N}}\beta_{i}f_{i}({\text{w}}_{i}{\text{x}}_{j}+b_{i})={\text{t}}_{j},j=1,\ldots ,N$$

Which can be simplified as:

(11) Hβ=T

where

(12) $$\text{H}({\text{w}}_{1},\ldots ,{\text{w}}_{\hat{N}},b_{1},\ldots ,b_{\hat{N}},{\text{x}}_{1},\ldots ,{\text{x}}_{N})={\left[\begin{array}{ccc} g({\text{w}}_{1}{\text{x}}_{1}+b_{1}) & \cdots & g({\text{w}}_{\hat{N}}{\text{x}}_{1}+b_{\hat{N}})\\ \vdots & \ddots & \vdots \\ g({\text{w}}_{1}{\text{x}}_{N}+b_{1}) & \cdots & g({\text{w}}_{\hat{N}}{\text{x}}_{N}+b_{N}) \end{array}\right]}_{N\times \hat{N}}$$

(13) $$\beta ={\left[\begin{array}{c} \beta_{1}^{T}\\ \vdots \\ \beta_{\hat{N}}^{T} \end{array}\right]}_{\hat{N}\times m},\text{T}={\left[\begin{array}{c} {\text{t}}_{1}^{T}\\ \vdots \\ {\text{t}}_{N}^{T} \end{array}\right]}_{N\times m}$$

ELM finishes the training within three steps. Firstly, the hidden weight and bias are randomly initialized, and their values remain frozen during the training. Then, the output matrix H is computed using training set. Finally, the output weight is computed from Eq. (11) by Moore–Penrose pseudo inverse:

(14) $$\beta ={\text{H}}^{†}\text{T}$$

where ***H***^†^ represents the pseudo inverse.

### 4.3 BAC

ELM is trained fast, and its classification performance is promising. However, random parameters in hidden layer have a bad effect on the robustness of ELM (Sun 2018). Therefore, ELM can be improved by further parameter optimization. In this paper, we employed BAC to optimize the hidden weight and bias in ELM. BAC is an improved form of bat algorithm (Yang 2010), which initializes a set of bat particles to search the solution space. Each bat particle contains a potential solution of the ELM parameters, moves with certain velocity and searches using ultrasound. In every iteration, the values of fitness function of all the bats will be computed and the parameters in bats will be updated according to the best solution obtained so far. There are some other swarm intelligence methods (Wu 2011a, b) which we will test in our future studies.

The introduction of chaotic map to the bat algorithm aims to enhance the randomness of bats to help them jump out of local extrema and reach the global optimized solution. There are various chaotic map functions, like Gaussian chaotic map, Logistic chaotic map and cubic chaotic map (Arasomwan and Adewumi 2014). The functions are as follows.

**Fig. 7:**
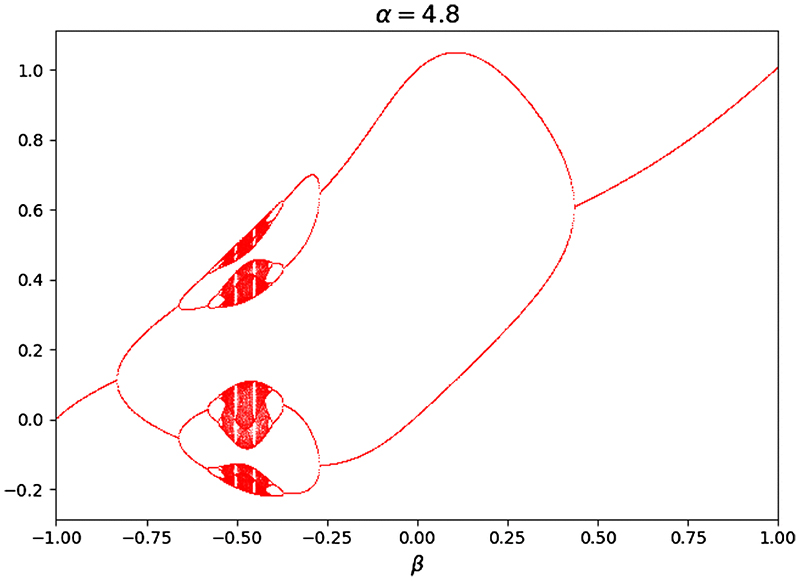
Diagram of Gaussian map

- Gaussian map
  (15) $$x_{k+1}=\exp(-\alpha x_{k}^{2})+\beta$$
  where *α* and *β* are two real parameters, and *k* represents the iteration time. The bifurcation diagram of Gaussian chaotic map is given in Fig. 7. The discrete form of Gaussian map can be expressed as
  (16) $$x_{k+1}=\{\begin{array}{rr} 0, & x_{k}=0\\ \frac{1}{x_{k}}-\left\lfloor \frac{1}{x}\right\rfloor , & x_{k}\in (0,1) \end{array}$$
  where ⌊*x*⌋ denotes the largest integer no more than *x*.
- Logistic map
  (17) $$x_{k+1}=rx_{k}(1-x_{k})$$
  where r represents the positive integer parameter.
- Cubic map
  (18) $$x_{k+1}=3x_{k}(1-x_{k}^{2})$$

**Fig. 8:**
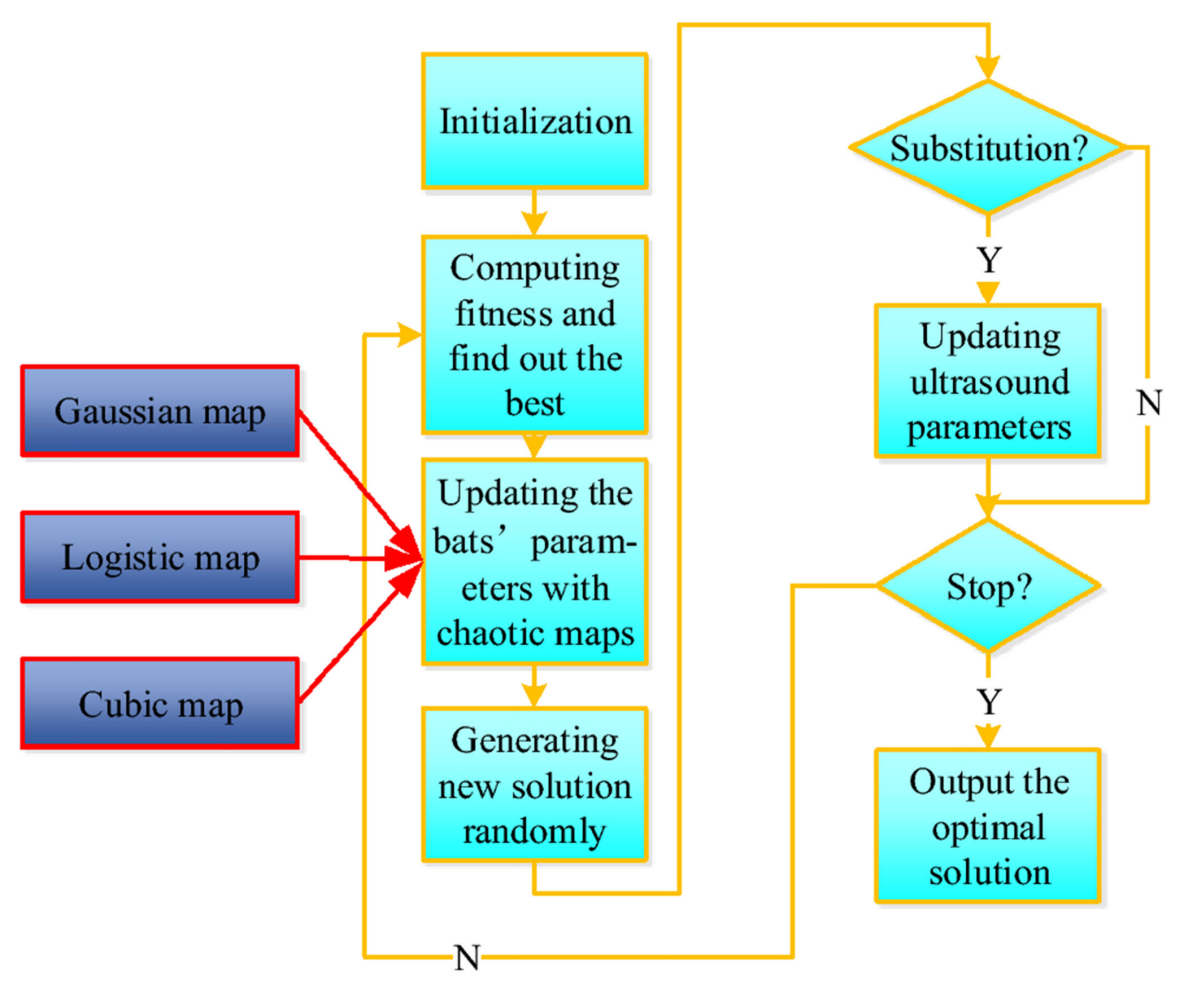
BAC algorithm

The flowchart of BAC is provided in Fig. 8. Firstly, all the bats are initialized randomly. Then, the fitness values of bats are computed, and the best solution is obtained among the bats. The positions of bats are updated according to the best solution so far along with chaotic map. The original bat algorithm updates the bats’ positions by

(19) $$x_{i}^{t}=x_{i}^{t-1}+v_{i}^{t}$$

where $v_{i}^{t}$ and $x_{i}^{t}$ denotes the velocity and position of bat *i* in the *t*^th^ iteration. In BAC, chaotic map was employed to enhance the randomness of bats to better explore the solution space, which was used in position updating:

(20) $$x_{i}^{t}=x_{i}^{t-1}+v_{i}^{t}+w\times \;\text{chaotic}\;(x_{i}^{t-1})$$

where w is a weighting parameter and is set as 0.3 in this study. Next, a new solution is generated around the best solution found by bats. If the newly generated solution performed better than the best solution found so far and the loudness of ultrasound is larger than a random generated value from [0,1], the new one will be accepted as the best solution and the ultrasound parameters will be updated. Afterwards, the programme will go back to calculate the bats’ fitness for iteration until it reaches the max iteration times.

Based on those three maps, we proposed three variants of BA: (1) Gaussian-map bat algorithm (GBA), (2) Logistic-map bat algorithm (LBA), and (3) Cubic-map bat algorithm (CBA).

### 4.4 Proposed methods

**Fig. 9:**
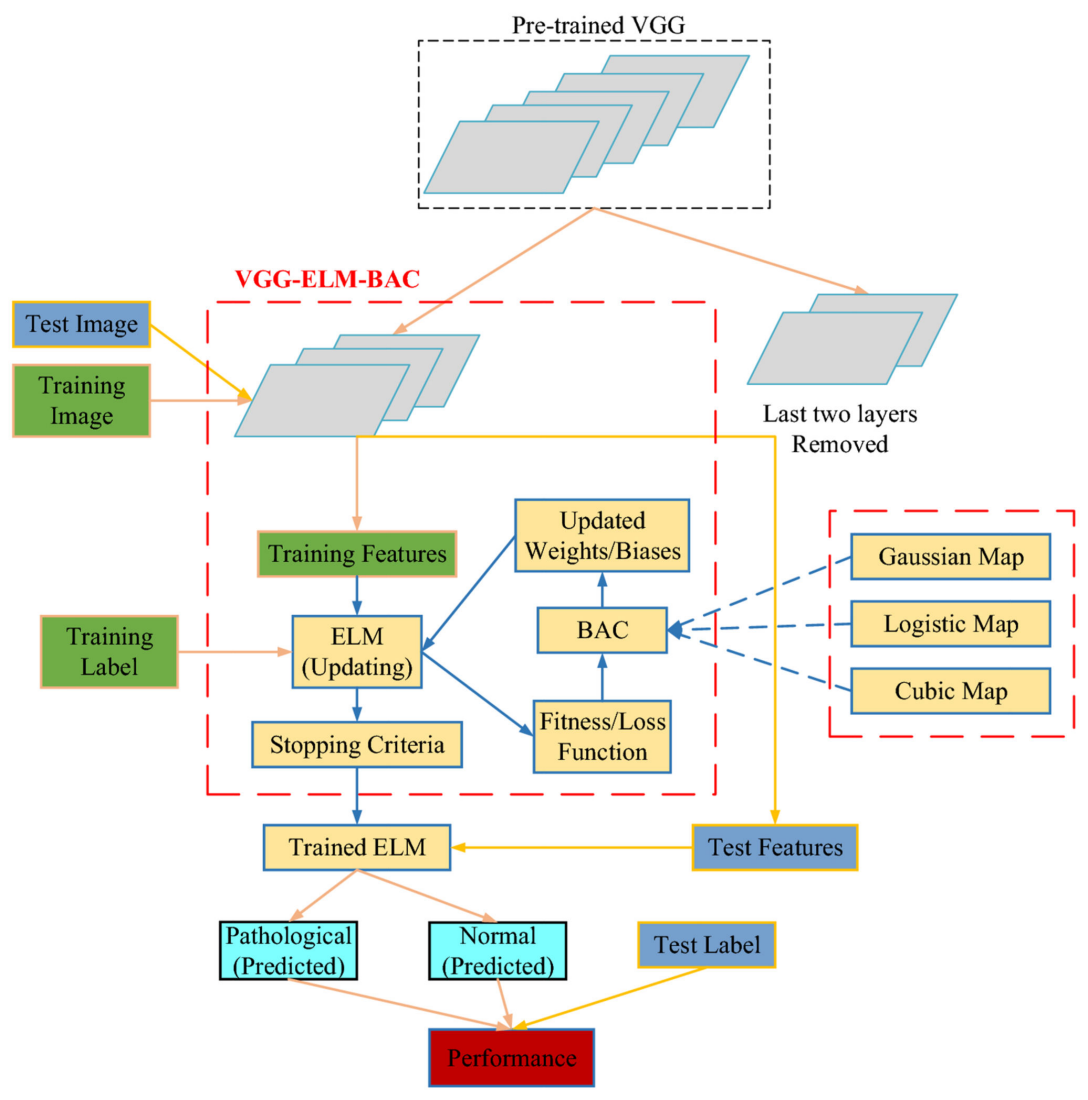
Flowchart of VGG-ELM-BAC

We proposed our VGG-ELM-BAC for CMB diagnosis. Firstly, a VGG was employed for feature extraction, which was pre-trained on a subset of ImageNet Large-Scale Visual Recognition Challenge (ILSVRC). We removed the last two layers before extracted its output as the image features. Then, the features were fed into an ELM for training. The hidden weights and bias of ELM were optimized by BAC algorithm. Finally, the trained model was evaluated on test set. The diagram of VGG-ELM-BAC was given in Fig. 9. We proposed three BAC methods: VGG-ELM-GBA, VGG-ELM-LBA, VGG-ELM-CBA.

## 5 Experiment

The proposed method VGG-ELM-BAC was developed on MATLAB 2018a with neural network toolbox. The system was run on a laptop with Intel i7 7700HQ CPU, 16 GB RAM, and NVIDIA GTX1060 GPU. We obtained totally 13031 samples in size of 41 × 41 in our dataset, with 6407 CMB and 6624 non-CMB, and 70% of the dataset is used for training set and the rest 30% for test set. Detailed information about the dataset is given in Table 2.

The hyper-parameters in our model are given in Table 3. The number of hidden nodes in ELM was set as 500, because the input space was 1000 × 1. We set the bats population as 20, considering the computational efficiency. The weight of chaotic and the parameters of bats were determined according to convention.

## 6 Results and discussion

### 6.1 Confusion matrix of proposed method

Our method achieved the best classification performance with the optimal feature layer ‘fc8’ and Gaussian map. The confusion matrix is presented in Table 4. We can calculate that the system achieved sensitivity of 93.08%, specificity of 87.12% and accuracy of 90.00%. Sensitivity is more significant in real application because it denotes the possibility of misclassifying a CMB sample into non-CMB. The patient may miss the valuable time and chance to get treatment.

### 6.2 Optimal feature layer

The feature layer denotes the layer in which the feature vector was extracted, and it is also the last layer reserved in VGG in our model. To get the optimal feature layer, an experiment was carried out. The results are illustrated in Table 5. The specificity of the four is relatively stable while the sensitivity varies. Obviously, the features from the ‘fc8’ layer performed better than features from other layers, so it is the best option. Meanwhile, the feature dimension from precedent layers will be larger than 4096, which is excessive to describe an image of 41 × 41 size.

### 6.3 Optimal chaotic map

We also tested three common chaotic maps to select the best for our system. The results are given below in Table 6. The “no map” means that the ELM was optimized by original bat algorithm without chaotic maps. The introduction of chaotic map can improve the classification performance of our system in terms of accuracy. Gaussian map achieved the best results on both sensitivity and accuracy. However, in terms of specificity, cubic and logistic map are better than Gaussian map. The Gaussian map has been shown to be a good example of a chaotic discrete dynamical system, which provided better chaotic mechanism to the bat algorithm than other maps so that the bat particles are more likely to get the global optimal solution. We choose Gaussian map because sensitivity is more important in our application.

### 6.4 Comparison with state-of-the-arts

**Fig. 10:**
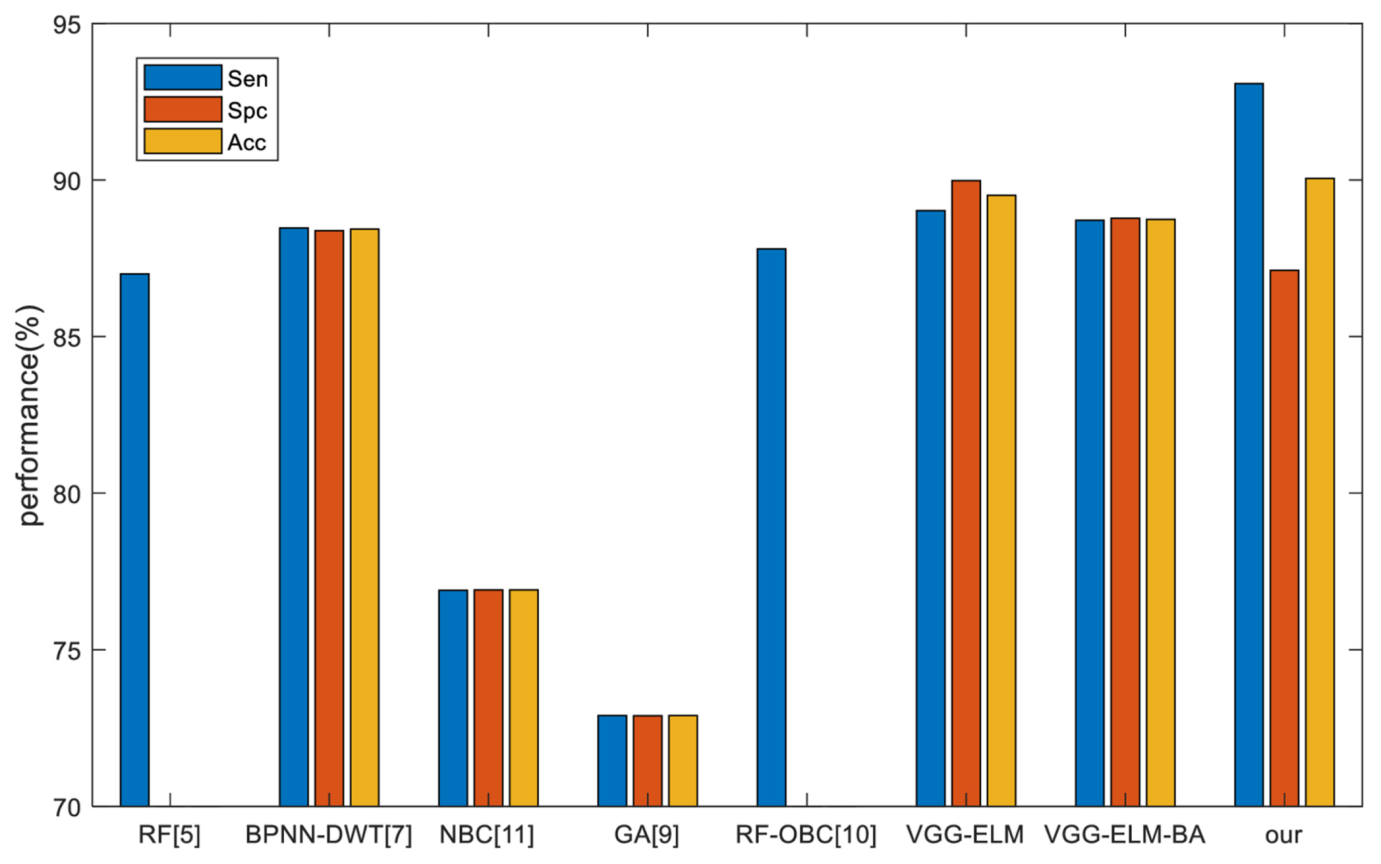
Comparison with state-of-the-art algorithms

We compared our VGG-ELM-GBA to state-of-the-art approaches: RF (Fazlollahi et al. 2015), BPNN-DWT (Hong and Lu 2019), NBC (Gagnon 2017), GA (Tao and Cloutie 2018), and RF-OBC (van den Heuvel et al. 2016). The results are provided in Table 7 and Fig. 10. We can see that our method achieved the best sensitivity and accuracy. For specificity, VGG-ELM outperformed just marginally. For medical applications, sensitivity is more significant because we hope to detect all the CMBs. The GBA optimization of ELM parameters can converge in 170.57 s, which is affordable in real application.

## 7 Conclusion

In this paper, a novel CMB diagnosis system was proposed based on VGG, extreme learning machine and bat algorithm with chaotic map. The trained system can distinguish CMB samples from non-CMB samples accurately, which provides a diagnosing reference for doctors.

However, it’s difficult to interpret how the diagnosis results are made. The model only solved a binary classification problem, but multi-class classification is more desired in practical application. The accuracy of the system is 90.05%, which is not perfect.

In the future, we shall collect more data and re-test the system. We shall try to apply our method to detect specific brain diseases and develop multi-class detection systems. Advanced deep models and structures can also be utilized in our future research, such as dilated convolution, and AlphaMEX Global Pool.

## Figures and Tables

**Table 1:** Layers in VGG-16 (C, MP and FC denote convolution, max pooling and full connected, respectively)

1	‘input’	Image input	Image size 224 × 224 × 3
2	‘conv1_1’	C	64 filters in size of 3 × 3 × 3, padding (1,1) and stride (1,1)
3	‘relu1_1’	ReLU	ReLU activation function
4	‘conv1_2’	C	64 filters in size of 3 × 3 × 3, padding (1,1) and stride (1,1)
5	‘relu1_2’	ReLU	ReLU activation function
6	‘pool1’	MP	Max pooling in size of 2 × 2, padding (0,0) and stride (2,2)
7	‘conv2_1’	C	128 filters in size of 3 × 3 × 64, padding (1,1) and stride (1,1)
8	‘relu2_1’	ReLU	ReLU activation function
9	‘conv2_2’	C	128 filters in size of 3 × 3 × 128, padding (1,1) and stride (1,1)
10	‘relu2_2’	ReLU	ReLU activation function
11	‘pool2’	MP	Max pooling in size of 2 × 2, padding (0,0) and stride (2,2)
12	‘conv3_1’	C	256 filters in size of 3 × 3 × 128, padding (1,1) and stride (1,1)
13	‘relu3_1’	ReLU	ReLU activation function
14	‘conv3_2’	C	256 filters in size of 3 × 3 × 256, padding (1,1) and stride (1,1)
15	‘relu3_2’	ReLU	ReLU activation function
16	‘conv3_3’	C	256 filters in size of 3 × 3 × 256, padding (1,1) and stride (1,1)
17	‘relu3_3’	ReLU	ReLU activation function
18	‘pool3’	MP	Max pooling in size of 2 × 2, padding (0,0) and stride (2,2)
19	‘conv4_1’	C	512 filters in size of 3 × 3 × 256, padding (1,1) and stride (1,1)
20	‘relu4_1’	ReLU	ReLU activation function
21	‘conv4_2’	C	512 filters in size of 3 × 3 × 512, padding (1,1) and stride (1,1)
22	‘relu4_2’	ReLU	ReLU activation function
23	‘conv4_3’	C	512 filters in size of 3 × 3 × 512, padding (1,1) and stride (1,1)
24	‘relu4_3’	ReLU	ReLU activation function
25	‘pool4’	MP	Max pooling in size of 2 × 2, padding (0,0) and stride (2,2)
26	‘conv5_1’	C	512 filters in size of 3 × 3 × 512, padding (1,1) and stride (1,1)
27	‘relu5_1’	ReLU	ReLU activation function
28	‘conv5_2’	C	512 filters in size of 3 × 3 × 512, padding (1,1) and stride (1,1)
29	‘relu5_2’	ReLU	ReLU activation function
30	‘conv5_3’	C	512 filters in size of 3 × 3 × 512, padding (1,1) and stride (1,1)
31	‘relu5_3’	ReLU	ReLU activation function
32	‘pool5’	MP	Max pooling in size of 2 × 2, padding (0,0) and stride (2,2)
33	‘fc6’	FC	Fully connected layer with 4096 nodes
34	‘relu6’	ReLU	ReLU activation function
35	‘drop6’	Dropout	Dropout probability 50%
36	‘fc7’	FC	Fully connected layer with 4096 nodes
37	‘relu7’	ReLU	ReLU activation function
38	‘drop7’	Dropout	Dropout probability 50%
39	‘fc8’	FC	Fully connected layer with 1000 nodes
40	‘prob’	Softmax	Softmax activation function
41	‘output’	Classification layer	Cross-entropy activation function

**Table 2:** Dataset configuration

Total samples			
13,031			
CMB		Non-CMB	
6407		6624	
Training		Testing	
9122		3909	
CMB	Non-CMB	CMB	Non-CMB
4485	4637	1922	1987

**Table 3:** Hyper-parameter settings

Hyper-parameter	Values
# of hidden nodes in ELM	500
# of population of bats	20
*w* for chaotic	0.3
Max iteration *i_max*	20
Max pulse loudness A_0_	1.6
Max pulse rate *R_0_*	1e-3
Loudness attenuation factor *α*	0.9
Frequency enhancement factor *γ*	0.99
Searching frequency range [*f*_min_, *f*_max_]	[0,2]

**Table 4:** Confusion matrix of our method

	Predicted label	
Actual label	Non-CMB	CMB
Non-CMB	1731	256
CMB	133	1789

**Table 5:** Performance of our system of different feature layers

Feature layer	Feature dimension	Sensitivity (%)	Specificity (%)	Accuracy (%)
Last 2nd layer: ‘prob’	1000	79.14	92.10	85.73
Last 3rd layer: ‘fc8’	1000	93.08	87.12	90.05
Last 4th layer: ‘drop7’	4096	79.50	89.08	84.37
Last 5th layer: ‘relu7’	4096	75.03	89.03	82.41

**Table 6:** Performance of our method with difficult chaotic maps

Chaotic map	Sensitivity (%)	Specificity (%)	Accuracy (%)
No map	88.71	88.78	88.74
Cubic	84.39	94.16	89.36
Logistic	89.02	89.83	89.43
Gaussian	93.08	87.12	90.05

**Table 7:** Performance comparison

Methods	Sensitivity (%)	Specificity	Accuracy
RF (Fazlollahi et al. 2015)	87.00	~	~
BPNN-DWT (Hong and Lu 2019)	88.47	88.38%	88.43%
NBC (Gagnon 2017)	76.90	76.91%	76.91%
GA (Tao and Cloutie 2018)	72.90	72.89%	72.90%
RF-OBC (van den Heuvel et al. 2016)	87.80	~	~
VGG-ELM	89.02	**89.98%**	89.51%
VGG-ELM-BA	88.71	88.78%	88.74%
VGG-ELM-GBA (our)	**93.08**	87.12%	**90.05%**
